# Re-assessment of re-warming for out-of-hospital births

**DOI:** 10.1186/s13049-020-00770-5

**Published:** 2020-08-05

**Authors:** Peter Jones, Camille Joly, Benoît Vivien

**Affiliations:** 1 SAMU de Paris, Hôpital Universitaire Necker - Enfants Malades, Assistance Publique – Hôpitaux de Paris, AP-HP, 149 rue de Sèvres, 75015 Paris, France; 2 PICU (Réanimation Pédiatrique), Hôpital Universitaire Robert Debré, Assistance Publique - Hôpitaux de Paris, AP-HP, 48 Boulevard Sérurier, 75019 Paris, France

**Keywords:** Newborn, Out-of-hospital, Hypothermia, Rewarming, Hyperthermia

## Abstract

Therapeutic controlled cooling is routinely practiced on neonates with core temperatures of 33–34 °C attained during cooling for birth related hypoxic-ischaemia encephalopathy (HIE). Rewarming after therapeutic cooling in clinical trials for HIE takes place at 0.25–0.5 °C/h over 6–12 h. Javaudin et al. looked at four methods for re-warming infants born out-of-hospital. The incubator group had a 0.8 °C median increase in body temperature for a median transfer time of 38 min (IQR-31-49 min); equating to 1.3 °C/h. In contrast, the group plastic bag+skin-to-skin+cap had a median temperature rise of 0.2 °C (median transport time 43 min [IQR-33-61 min]); equating to 0.28 °C/h, which is closer to therapeutic controlled methods. Javaudin et al. proposed incubator re-warming for out-of-hopital births whereas we consider that an alternative interpretation of the article’s results leads to the different conclusion that plastic bag+skin-to-skin+cap, rather than an incubator, is the preferable method due to the more progressive re-warming and lower frequency of hyperthermia.

Sir,

We were interested to read the article in the journal by Javaudin et al. [1] looking at the re-warming of infants born out-of-hospital. Whilst the authors are to be congratulated for a careful comparison of several different techniques, their conclusions should be interpreted with caution.

The authors’ hypothesis that hypothermia is an independent risk factor for neonatal mortality in *term* infants cannot be substantiated. Of the four articles cited in the text, one concerned premature births, two looked at low birth weight infants and the fourth looked at a group of mixed gestational ages (with prematurity being an associated with increased mortality).

Therapeutic controlled cooling is routinely practiced on neonates with core temperatures of 33–34 °C during cooling for birth related anoxo-ischaemia [2] and 26–28 °C attained during surgical correction of congenital heart disease.

Rewarming after therapeutic cooling in clinical trials for hypoxic-ischaemic encephalopathy (HIE) takes place at 0.25–0.5 °C/h over 6–12 h with seizures reported during too rapid re-warming [2].

Javaudin et al.’s incubator group had a 0.8 °C median increase in body temperature for a median transfer time of 38 min (IQR-31-49 min); equating to 1.3 °C/h. In contrast, the group plastic bag+skin-to-skin+cap had a median temperature rise of 0.2 °C (median transport time 43 min [IQR-33-61 min]); equating to 0.28 °C/h which is closer to therapeutic controlled methods.

Re-bound hyperthermia was limited in all groups studied but was present in 2.0% of the plastic bag+skin-to-skin+cap group and 3.1% in the incubator group, although the sample size is insufficient to permit a valid statistical comparison for these data.

Short periods of mild to moderate hypothermia have not been proven to increase mortality in term infants. However, hyperthermia has injurious effects on neurological and cognitive function [3]. As such, the principal objectives of rewarming infants born out-of-hospital should be the avoidance of both excessive cooling and rapid or excessive re-warming [4, 5]. Additionally, it should be born in mind that a proportion of these infants will have suffered from unquantified HIE during birth and it is not in their interest to arrive at hospital with higher core temperatures.

An alternative interpretation of the article’s results leads to the different conclusion that plastic bag+skin-to-skin+cap rather than an incubator is the preferable method of re-warming due to the more progressive re-warming and lower frequency of hyperthermia. Nevertheless, the authors are to be congratulated for their valid contribution in having marginalised the practice of rewarming by plastic bag+cap and skin-to-skin+cap.

## Data Availability

No data or materials were used in the preparation of the manuscript and as such none can be made available.

## Abbreviation

HIE: Hypoxic-ischaemic encephalopathy
